# Large area highly ordered monolayer composite microsphere arrays – fabrication and tunable surface plasmon linewidth

**DOI:** 10.1039/c8ra07564f

**Published:** 2018-11-27

**Authors:** Haibin Ni, Lu Ge, Xiang Liu, Ying Zhou, Jianhua Chang, Hassan Ali, Chao Pan, Tingting Wang, Ming Wang

**Affiliations:** Jiangsu Key Laboratory of Meteorological Observation and Information Processing, School of Electronics and Information Engineering, Nanjing University of Information Science and Technology Nanjing 210044 China nihaibin@nuist.edu.cn; State Key Laboratory of Bioelectronics, School of Biological Science and Medical Engineering, National Demonstration Center for Experimental Biomedical Engineering Education, Southeast University Nanjing 210096 China; Department of Biomedical Engineering, University of Engineering and Technology Lahore 60000 Pakistan; Jiangsu Key Laboratory on Optoelectronic Technology, School of Physical Science and Technology, Nanjing Normal University Nanjing 210023 China

## Abstract

A route to produce highly ordered two-dimensional periodic composite microsphere/gel arrays by using a sol–gel coassembly method was proposed and demonstrated. The proposed semi-infiltrated ordered monolayer PS microsphere/gel array affords a flexible platform to produce versatile plasmonic structures through either adjusting the gel infiltration height or sputtered metal film thickness. Fabrication factors, such as environmental humidity, evaporation temperature, and tetraethyl orthosilicate solution concentration, that will affect the quality of the monolayer film were experimentally investigated. Pair correlation function was applied to evaluate the order degree of the experimental results, which reveals the high uniformity of the composite microsphere arrays (CSAs). By adjusting metal film thickness, the figures of merit of propagating surface plasmons excited on CSAs or concave arrays can be tuned under normal incidence.

## Abstract 2

Surface plasmons on co-assembled large area highly ordered monolayer composite sphere arrays exhibit tunable linewidth.

## Graphical abstract

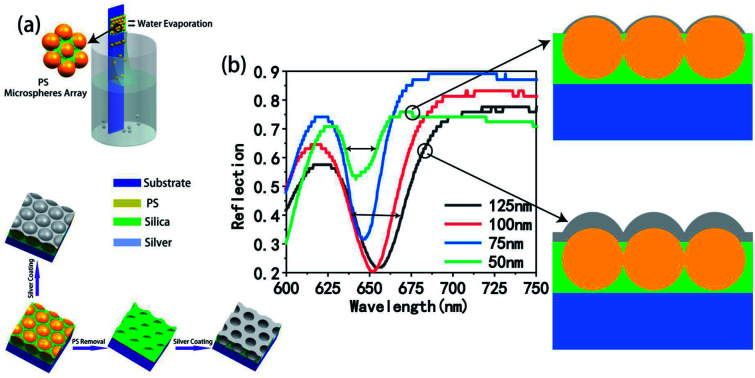

## 1. Introduction

Surface plasmons (SP) have been widely explored in sensing,^1–4^ wavefront shaping,^5–7^ waveguides,^8–11^ and particle trapping^12–15^ since they have the ability to break the diffraction limits. As one of the most popular applications of SPs, SP sensors attract much attention in the environmental and bio-sensing area. While high sensitivity and figures of merit (FOM) assure reliable sensing performance,^2,16–18^ miniature size, good reproducibility, and high fabrication efficiency are also required for the widespread application.

Two-dimensional (2D) periodic metal nanostructures comprise an effective approach to excite propagating surface plasmons. Propagating SPs can be easily excited at the interface of these structures at normal or tilt incidence by linearly polarized light, which is promising for miniature size or integrated SPP based sensors.^17,18^ Among versatile nanofabrication methods, self-assembly of monolayer periodic structures proves to be an easy approach to create periodic patterns that can support SPs in visible and near infrared range, which requires unit structure size in the range from tens to hundreds of nanometers.^20^ And the order degree of the microsphere arrays is vital as propagating SP is dependent on the periodicity of the structures.^3,21,22^ It is worthy to note that besides the propagating SP mode, 2D periodic patterns can also be applied to study the localized surface plasmon (LSPR) mode for some materials that usually are hard be prepared in nanoscale, such as VO_2_ nanostructures with temperature tunable LSPR.^19^

It is known that producing long-range highly ordered monolayer microsphere arrays by self-assembly method is always a challenge. Great efforts have been devoted to producing highly ordered monolayer microsphere arrays in a large area with high efficiency in previous, and many methods were proposed, including spin coating,^23–27^ air–liquid or liquid/liquid interface^28–31^ and two substrates selfassembly,^32^*etc.* Air–liquid interface self-assembly method could form large area monolayer microsphere arrays but remains with imperfection in the film such as over layered microspheres or small single domain area, while spin coating was constrained by low order degree over a large area and two substrates self-assembly is time-consuming and also cannot form non-crack, highly ordered monolayer microsphere arrays. As a result, none of those methods can produce highly ordered arrays over a large area. However, the lifetime of SPP is largely dependent on the periodicity of microspheres, which determines the linewidth of the propagating SP mode and further affect the FOM.

Inspired by Hatton,^33^ we managed to produce ever highly ordered monolayer composite microsphere arrays (CSAs) over centimeter length. Take advantages of silica gel and meniscus at air/liquid/substrate interface with optimized parameters, microspheres are arranged in high quality over a large area with only fewer domain borderlines, which could largely decrease the linewidth of the propagating SPs. The order degree of CSAs was characterized by the pair correlation function. In addition, due to the semi-infiltrated silica gel, the loss of the propagating SPs increases as the thickness of sputtered metal film increase, which is attributed to the increased gap depth.^17,18^ Thus, tunable FOM can be obtained *via* control of the deposited metal film thickness.^2,17^ Furthermore, periodic Ag hemispherical concave arrays produced from CSA also exhibit both tunable SP wavelength and linewidth by varying the height of infiltrated silica gel and coated metal film thickness.

## 2. Experimental

### 2.1. Preparation of monolayer composite PS microsphere arrays

**Fig. 1:**
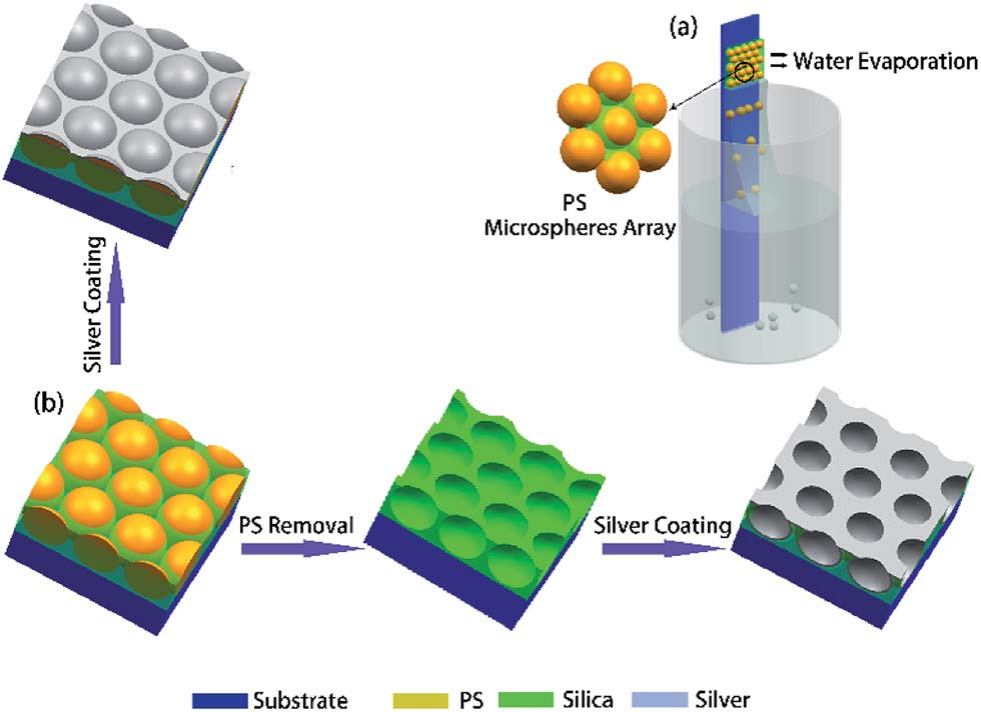
(a) Schematic diagram of the setup to vertical self-assembly of monolayer composite microsphere array on a substrate. (b) Typical fabrication steps to produce Ag hexagonal periodic semi-sphere patterns and Ag hexagonal periodic concave patterns.

Schematic diagram of the self-assembly of CSA was illustrated in Fig. 1a. The vial containing monodisperse polystyrene (PS) microsphere aqueous solution (Bangs Laboratory Inc., 10%vol) with a diluted concentration of 0.05%vol to 0.2%vol, and diameter of the PS spheres are ranging from 250 nm to 690 nm. An amount of acid catalyzed tetraethyl orthosilicate (TEOS) solution (∼0.01%vol) was also added to the vial and mixed with PS aqueous solution by ultrasonic for 5 min. The acid catalyzed TEOS solution was prepared by mixing tetraethyl orthosilicate (98%wt), 0.1 mol L^−1^ HCl, and absolute ethanol (100%) with a weight ratio of 1 : 1 : 1.5 and stirring for 1 h before use. Slides were used as substrates for the assembling of monolayer composite microsphere/silica gel films. Before using they were cleaned with ultrasonic bath with water, acetone (99.9%), ethanol (99.9%) for 5 min, respectively, and then washed with deionized water (18.25 MΩ) for 2 min. And then these slides were immersed in a piranha solution (of H_2_O_2_/H_2_SO_4_ with a volume ratio of 3 : 7) at 80 °C for 3 h, and finally washed with deionized water for 2 min and dry with nitrogen. The cleaned substrate was vertically inserted into the glass vial and partially immersed in the solution and fixed (see Fig. 1a). Finally, the solution was placed in an oven with a constant temperature (Zhi Cheng instruments) and relative humidity. A meniscus is formed at the interface of air, solution, and the hydrophilic substrate in the vial. As solvent evaporated in the meniscus and the liquid surface, PS microspheres in the solution were continuously transported to the meniscus, and capillary force-driven microspheres to accumulate in the meniscus and the silica precursor solution are filled in the gaps between microspheres in the form of a gel as solvent evaporated. After about 24 h as the solvent was evaporated, a monolayer of composite microspheres array can be obtained on the slide.

Followed by sputtering a layer of Ag film on the self-assembled CSA, we obtained the Ag periodic semi-sphere patterns, as shown in Fig. 1b. The deposition rate of Ag film is ∼0.4 Å s^−1^ which was monitored by a quartz oscillator.

### 2.2. Preparation of periodic Ag hemispherical concave arrays

To produce periodic Ag hemispherical concave arrays, the PS microspheres in CSA were removed by sintering at 500 °C or dissolving in toluene for two hours. Then, sputtering a layer of Ag film on it to obtain the periodic Ag hemispherical concave arrays, as shown in Fig. 1b.

### 2.3. Preparation of monolayer PS microsphere array

Monolayer PS microspheres (250 nm, Bangs Laboratory Inc.) were dispersed in aqueous/ethanol (volume ratio 1 : 1) mixed solution with a concentration of 2%vol. The mixed solution was injected by a string to the air/water interface with a speed of 0.2 μL s^−1^. After a layer of PS microsphere array was formed at the liquid surface, than transfer it to a glass substrate and dry slowly in air.

### 2.4. Characterization

The top view and cross-section morphology of CSAs were characterized by scanning electron microscope (SEM) (JEOL7600). The quality of the CSA films in a large area was characterized by an optical microscope (Nikon L150). White light ranging from 400 nm to 1800 nm (Yokogawa AQ4305) was coupled to an optical fiber and was impinged to the surface of CSA films, and the reflected light was collected by optical fibers and measured by an optical spectrum analyzer (Ocean Optics, USB 2000).

## 3. Results and discussion

### 3.1. Morphology of CSA and fabrication parameters optimization

**Fig. 2:**
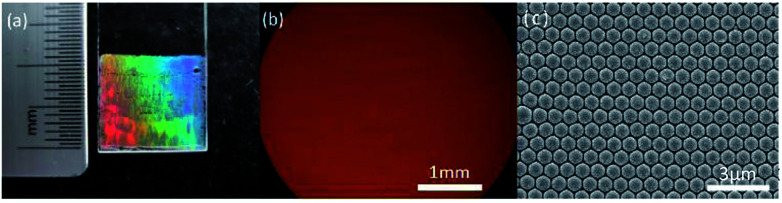
Monolayer highly ordered CSAs on a slide. (a) Camera image, (b) optical microscope image, (c) SEM image.

Large area (more than 1 cm × 1 cm), highly ordered monolayer CSAs was created by the coassembly method with optimized fabrication parameter, as shown in Fig. 2. In experiments, the evaporation rate of the solvent determines the quality of self-assembled CSAs while temperature and humidity are two main factors that determine the evaporation rate. In order to obtain the optimized temperature and relative humidity for high-quality CSAs, varied evaporation temperature and relative humidity have been used in the experiments.

**Fig. 3:**
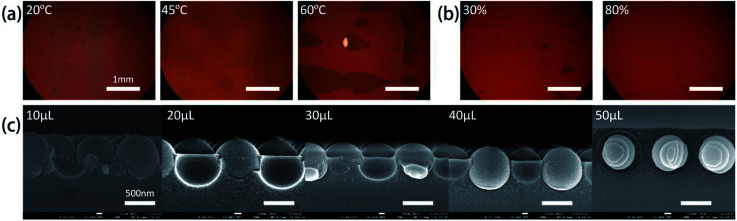
(a) Optical microscope images of CSAs film with different evaporation temperature at 60% RH (20 °C, 45 °C and 60 °C, from left to right). (b) Optical microscope images of CSAs film produced with different relative humidity at 45 °C (low relative humidity (∼30%), high relative humidity (∼80%), from left to right). (c) SEM images of CSAs produced from 5 mL solution added with different volume of TEOS solution (10 μL, 20 μL, 30 μL, 40 μL, and 50 μL, from left to right).

Three temperature 20 °C, 45 °C, 60 °C have been used in the experiments while the relative humidity was set at about 60%. Fig. 3a shows that lower temperature (20 °C) leads to less kinetic energy for the microspheres to concentrate at the interface line during evaporation,^34^ which results in a low-quality film with small domain area. As the temperature increases, solvent evaporation rate also increases, and if the speed of microspheres assembled at the interface line equals to the liquid recede speed at the interface of air/liquid/substrate, the microspheres will form a high-quality CSAs film. Then a relatively good quality film is formed at 45 °C. At this temperature, evaporation speed is not so fast and microspheres have enough time to migrate and find the proper position and thus forming defect free monolayer CSAs in a large area. Evaporation temperature at 60 °C leads to an exceedingly large evaporation rate at the meniscus, and thus microspheres tend to have less time to find the thermodynamically stable lattice position before the solvent evaporated at the meniscus. In addition, higher temperature tends to form bi-layers due to the fast transportation of microspheres.

Besides temperature, relative humidity was also optimized in the fabrication process. Fig. 3b shows optical micrograph images of samples prepared under two different relative humidity of 30% and 80% when the temperature is set at 45 °C. As a comparison, the sample with the higher relative humidity of 80% exhibits a larger single crystal area and fewer vacancy defects. Meanwhile, the sample prepared at lower relative humidity shows a poor quality film, evidenced by several spots of bio-layers of microspheres.

Decrease relative humidity may increase the lateral capillary force between microspheres at the meniscus and thus increase the friction force between the substrate and the microspheres which suppressing the rearranging process inside the array and leads to the small domain.^34,35^ In addition, lower relative humidity increases the evaporation rate and creates more cracks and defects in the film. However, relative humidity higher than 80% results in very slow assembly speed and lower friction between substrate and arrays, and thus the film is more likely to be influenced by environmental vibrations.

Another factor, TEOS concentration, also plays a vital role in the morphology of CSAs.^36–38^ The effect of the added volume of TEOS solution on the quality of the film was given in Fig. 3c. For a fixed volume of PS aqueous solution and concentration of PS microspheres, increased concentration of TEOS solution results in different infiltration height. In a wide range, the TEOS concentration does not affect the infiltration height significantly but there is an upper and lower threshold of the concentration for a dramatic change of the infiltration height. Fig. 3c shows the cross sections of CSA film prepared at different TEOS concentrations. Lower TEOS solution concentration (<10 μL TEOS solution) leads to the low height and non-uniformity infiltration while higher TEOS concentration (>50 μL TEOS solution) results in an over layer on the PS microspheres. In the self-assembly process, the final silica gel infiltration height is determined by the meniscus between neighboring microspheres as long as the TEOS concentration is above the lower threshold value and below the higher threshold.

### 3.2. Order of the microsphere arrays

**Fig. 4:**
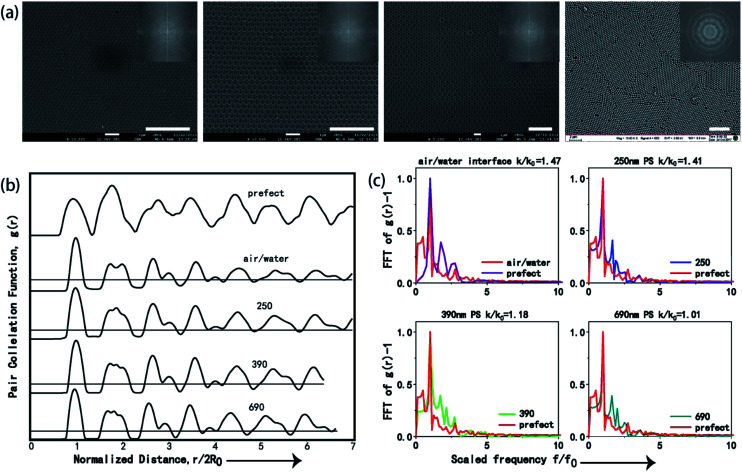
(a) SEM images of the CSAs fabricated from three different size PS microspheres by co-assembly method and monolayer PS microsphere arrays produced by air/water interface self-assembly method (from left to right: 250 nm PS microspheres self-assembled at air/water interface, and 250 nm, 390 nm and 690 nm PS microspheres by co-assembly method), the scale bar is 2 μm. The inset is the FFT of the image. (b) Pair correlation function, *g*(*r*), of CSAs from 250 nm, 390 nm, 690 nm PS microspheres and monolayer PS microsphere arrays produced by air/water interface self-assembly method. Horizontal black lines indicate *g*(*r*) = 1. (c) Single-sided power spectra Fourier transforms (FT) of *g*(*r*) compared to FT of the corresponding perfectly ordered arrays. The power spectra were scaled to have identical maxima at *f*/*f*_0_ = 1.

To quantitatively evaluate the order degree of the produced 2D CSAs, both fast Fourier transformation (FFT) and pair correlation function *g*(*r*) were applied to analyze the SEM images, as shown in Fig. 4a. Monolayer PS sphere array from 250 nm PS at air/liquid interface was used as a comparison with CSAs assembled from 250 nm, 390 nm, and 690 nm PS spheres. FFT has peaks at spatial frequencies of repeated structures. Image of long range, a high order of the sphere array will result in a sharp point pattern after FFT while disorder or short-range order will result in blur or even no sharp point pattern. Here, periodic sharp point patterns generated by FFT of the SEM image of CSA (Fig. 4a inset) confirms the long-range highly ordered periodic sphere arrays. As a comparison, FFT of SEM image of PS microsphere array self-assembled at air/water interface exhibit a blur pattern because of small domain area and disorder, as shown in the last image inset of Fig. 4a.

Furthermore, we performed the two-dimensional radial distribution analysis of the SEM images of CSAs by using the dimensionless pair correlation function, *g*(*r*), as shown in eqn (1):^3,21,22^1
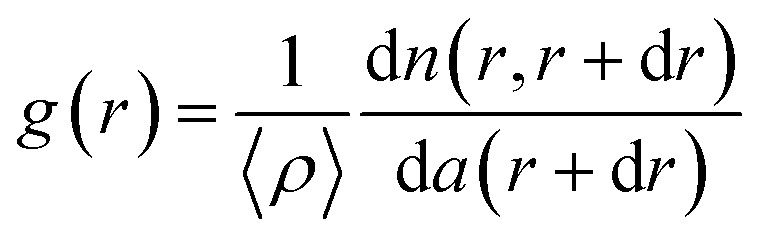
where *a* is the shell area and *n*(*r*, *r* + d*r*) is the number of microspheres that lie within the shell considered. *g*(*r*) calculated from different CSAs with varied sphere diameter and monolayer PS microsphere array were compared with a perfect hexagonal array, as shown in Fig. 4b. *R*_0_ represents the radius of the microsphere, the peaks means that the probability of finding a microsphere at this point is maximum. It can be observed that the positions of these peaks of CSAs and the monolayer PS microsphere arrays were substantially consistent with the perfect array, confirming the high-quality order of these arrays.

Fig. 4c shows the Fourier transform of the function *g*(*r*) − 1 for the ideal perfect array and four fabricated arrays. *k* is the full width at half maximum (FWHM) of the first peak, and *k*_0_ corresponds to that of the perfect array. Normalized ratio between *k* and *k*_0_, which is *k*/*k*_0_, is applied to evaluate the minor difference between *g*(*r*), and thus *k*/*k*_0_ = 1 suggests an ideal array, an increase of disorder in the structure can increase the value of *k*/*k*_0_, and the value of *k*/*k*_0_ > 1.5 indicates significant disordering of the structure.^3,21,22^ The *k*/*k*_0_ values for CSAs with PS microspheres of 250 nm, 390 nm, 690 nm, and monolayer PS microspheres (250 nm) self-assembled at air/water interface were calculated to be 1.41, 1.19, 1.01, and 1.47, respectively, which clearly show that the ordering of the 690 nm CSA is the best. As a comparison, though the PS microsphere arrays self-assembled at air/water interface are ordered structure, the order degree of them is the worst according to *k*/*k*_0_. Hence, the co-assembly method used in our work is superior to the air/liquid interface self-assembly method, which is widely used to produce monolayer ordered sphere arrays.

Besides PS spheres, spheres of other materials that can suspension in water can also be applied in this method. But physical property difference between the sphere and the gel should be considered as it will affect the following process to remove the sphere or other morphology modification.

### 3.3. Optical properties of thin Ag film on CSA

It is known that the Bloch wave surface plasmon polaritons (BW-SPPs) as a propagating SP can be excited on periodic metal/dielectric interface. For CSAs coated with a layer of Ag film, BW-SPPs can be excited and predicted, the relation for the incident wavelength of a BW-SPP (*λ*_spp_) can be described by the following equation:^2,17^2
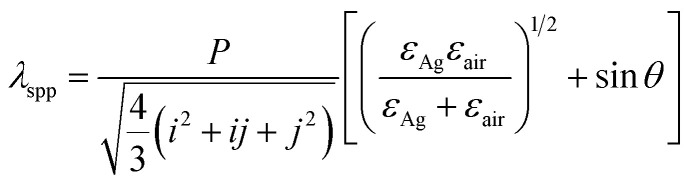
where *P* is the periodicity of the array, *i*, *j* are the orders of diffraction, *θ* is the incident angle, *ε*_Ag_ and *ε*_air_ are the dielectric functions of Ag and air, respectively. Here, *P* of CSA is 690 nm since the microspheres are close-packed.

Besides wavelength of BW-SPP which can be tuned by adjusting the size of PS microspheres (*P*), metal film (*ε*_Ag_), surrounding dielectric constant (*ε*_air_), and incident angle (*θ*), according to eqn (2), the line width of the BW-SPP is also a key property to be considered. The lifetime (*τ*) of the SPP mode is associated with the homogeneous linewidth of BW-SPP^17,39^ through *Γ* = 2*ℏ*/*τ*, where *Γ* is the FWHM of the BW-SPP mode. Hence, we can increase the lifetime *τ* to achieve minimum FWHM since FWHM is of significant importance on the performance of BW-SPP based sensors.

Previous research reveals^17,18^ that inherent nonradiative (ohmic) losses of the metal film and radiative losses due to evanescent SPP modes into the far field contribute to the reduction of *τ*. To increase *τ*, then we can decrease one or both of the above losses. Here, we demonstrate a convenient approach to tune the losses of metal film and thus change the lifetime *τ* of BW-SPP by adjusting the depth of gaps between neighboring semispheres, which eventually can tune the FOM.

**Fig. 5:**
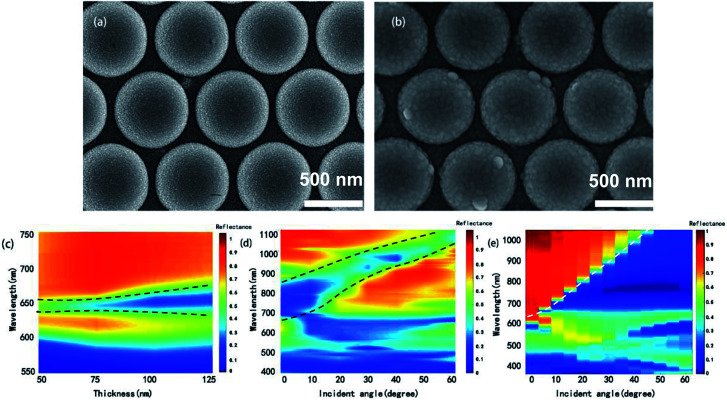
SEM images of CSAs without (a) and with 75 nm silver film (b). (c) Experimentally measured reflectance spectra of CSAs with silver film thickness increased from 50 nm to 125 nm. (d) Simulated and (e) experimentally measured angle-dependent reflectance spectra from 0° to 60°.

Our discussion will mainly focus on (1, 0) Ag/air BW-SPP as its corresponding reflectance dip can be clearly distinguished from other dips in measured reflectance spectra. Two structures with tunable BW-SPP line width are demonstrated here. One is CSAs coated with a layer of silver film and another one is periodic Ag semispherical concave arrays. When coating a layer of metal film, morphology of the top surface of CSA will slightly change, and if the metal film thickness continuous to increase, the depth of gaps between neighboring spheres will slightly increase and result in a change of the propagation loss of SP excited on the metal film. SEM images of CSAs with and without Ag film are given in Fig. 5a and b. Fig. 5c shows experimentally measured reflectance spectra of CSAs with an increased silver film thickness from 50 nm, 75 nm, 100 nm, and 125 nm, respectively. It can be seen that FWHM of the BW-SPP mode at ∼650 nm increased as the silver film thickness increase while the location of the reflection peak remains unchanged. The FWHM increases about 24% as film thickness increased from 50 nm to 125 nm, which is due to the increase of radiative loss induced by increased gap depth. Since the periodicity of the arrays is fixed, the BW-SPP mode of these arrays are nearly at the same wavelength and show same sensitivity, therefore, we can tune the FOM of them by varying the deposited metal film thickness.

In addition, we also investigate the angle-resolved properties of the CSA, which also can change the FWHM and further confirm this reflectance dip is attributed to (1, 0) Ag/air BW-SPP mode. As revealed by eqn (2), the value of *λ*_spp_ shifts to longer wavelength as the angle of incident light increases, and the finite-difference-time-domain (FDTD) simulated results are in coincidence with the theory prediction (Fig. 5d). Moreover, as the angle of incident light increases from 0° to 60°, the FWHM of the (1, 0) SPP mode decrease about 26%. Experimentally measured angle-resolved reflectance spectra further confirm that the reflectance dip at ∼650 nm is related to (1, 0) SPP mode. However, different from the simulated results in Fig. 5d, the dip at ∼650 nm also broadened as it shifts to longer wavelength in measured reflectance spectra, as plotted in Fig. 5e, which probably attributed to the excitation of localized surface modes on each metal semisphere shell on the top surface of CSA.

As a result, either increase incident light angle or decrease the metal film thickness, FWHM can be reduced. And thus higher FOM can be obtained, which is vital in bio-sensing applications, such as the trace detection of special gases^40^ and detecting bovine serum albumin with an ultralow concentration.^17^

### 3.4. Periodic Ag hemispherical concave arrays

**Fig. 6:**
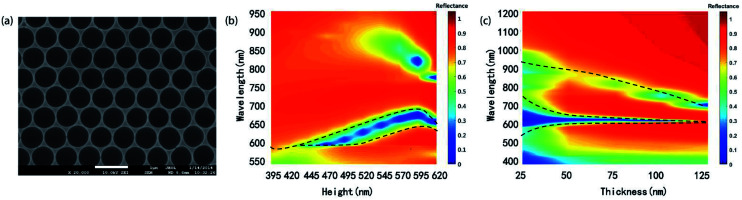
(a) SEM image of periodic hemispherical concave array. (b) Reflectance spectra of the concave array with different gel infiltration thicknesses. (c) Reflectance spectra of concave array with the silver film thickness increased from 25 nm to 125 nm.

Another structure, periodic Ag hemispherical concave arrays, (Fig. 6a) can be produced from CSA by removing PS microspheres and metal film coating, which have been an attracting structure for LED and solar cell applications. Role of the height of gel (*H*_c_) and the silver film thickness (*d*) on the reflectance spectra was discussed.

We use FDTD algorithm to simulate the reflectance spectra of the concave arrays as the *H*_c_ gradually increases from 395 nm to 620 nm while *d* was maintained at 50 nm. The refractive indices of PS were assumed to be 1.56, and a spatial mesh size of 5 nm (Δ*x* = Δ*y* = Δ*z*) is applied in the simulation area.

As shown in Fig. 6b, as *H*_c_ increases, the resonance wavelength of (1, 0) SPP mode redshifts and the reflectance dip grow narrow, which is partly due to the electron density change of the metal surface.^2,16^

Silver film thickness also plays an important role in the reflectance spectra for the concave array. We calculated reflectance spectra of concave arrays (*H*_c_ = 520 nm) with different silver film thicknesses of 25 nm, 50 nm, 75 nm, 100 nm, and 125 nm respectively, as exhibited in Fig. 6c. By increasing film thickness *d*, FWHM of (1, 0) Ag/air peak decreases by about 50% while the peak wavelength located at 650 nm do not shift obviously. The decrease of FWHM can be explained by the coupling effect between (1, 0) Ag/air and cavity mode. The cavity mode at 900 nm shifts to a shorter wavelength as metal film thickness increase. Moreover, the geometry size of the concave array can be controlled in the fabrication process, which affords more freedom on spectra tuning for specific applications.

## 4. Conclusions

We have developed a method to produce highly ordered CSAs using self-assembly methods. Morphology of CSAs can be adjusted by precisely adjusted experiment parameters. Consists of periodic PS microspheres and silica gel, CSAs not only exhibit tunable SPP lifetime (or FOM) by adjusting deposited metal film thickness but also can be used to produce concave arrays with tunable cavity mode. Furthermore, CSA also shows great potential to expand to other kinds of plasmonic applications.

## Conflicts of interest

The authors declare no conflict of interest.

## Supplementary Material
